# Graph Neural Networks in Brain Connectivity Studies: Methods, Challenges, and Future Directions

**DOI:** 10.3390/brainsci15010017

**Published:** 2024-12-27

**Authors:** Hamed Mohammadi, Waldemar Karwowski

**Affiliations:** Computational Neuroergonomics Laboratory, Department of Industrial Engineering and Management Systems, University of Central Florida, Orlando, FL 32816, USA; wkar@ucf.edu

**Keywords:** graph neural networks (GNNs), brain connectivity, neuroimaging, interpretability multimodal data integration, neurodegenerative diseases

## Abstract

Brain connectivity analysis plays a crucial role in unraveling the complex network dynamics of the human brain, providing insights into cognitive functions, behaviors, and neurological disorders. Traditional graph-theoretical methods, while foundational, often fall short in capturing the high-dimensional and dynamic nature of brain connectivity. Graph Neural Networks (GNNs) have recently emerged as a powerful approach for this purpose, with the potential to improve diagnostics, prognostics, and personalized interventions. This review examines recent studies leveraging GNNs in brain connectivity analysis, focusing on key methodological advancements in multimodal data integration, dynamic connectivity, and interpretability across various imaging modalities, including fMRI, MRI, DTI, PET, and EEG. Findings reveal that GNNs excel in modeling complex, non-linear connectivity patterns and enable the integration of multiple neuroimaging modalities to provide richer insights into both healthy and pathological brain networks. However, challenges remain, particularly in interpretability, data scarcity, and multimodal integration, limiting the full clinical utility of GNNs. Addressing these limitations through enhanced interpretability, optimized multimodal techniques, and expanded labeled datasets is crucial to fully harness the potential of GNNs for neuroscience research and clinical applications.

## 1. Introduction

In recent years, advancements in neuroimaging and computational modeling have transformed our understanding of the human brain as a complex network, where brain connectivity serves as a foundation for cognition, behavior, and neurological health [1]. Brain connectivity reflects the structural and functional pathways that facilitate communication between distinct regions, and its analysis provides insights into how neural circuits process information and adapt in response to experiences and changes in the environment [2]. Alterations in these pathways are often associated with a range of neurological and psychiatric conditions, including Alzheimer’s disease, autism spectrum disorder, and major depressive disorder, underscoring the importance of studying brain connectivity to develop effective diagnostic, prognostic, and therapeutic approaches.

Traditional approaches to studying brain connectivity—such as structural imaging with diffusion tensor imaging (DTI) and functional imaging with functional magnetic resonance imaging (fMRI) and electroencephalography (EEG)—have provided foundational insights into static and dynamic connectivity patterns [1]. These techniques have revealed connectivity as a multi-dimensional construct, comprising structural, functional, and effective components, each contributing uniquely to our understanding of the brain’s network architecture. However, they are limited by the complexity of human brain networks, which contain high-dimensional, non-linear relationships that often cannot be captured fully with conventional statistical or graph-theoretical models [3].

A key advancement has been the application of graph-based methods to brain connectivity, where brain regions are represented as nodes and their connections as edges, thus enabling the characterization of connectivity patterns through graph-theoretical metrics. This approach allows for a quantification of network properties, such as clustering and path length, the identification of network hubs, and the examination of dynamic connectivity changes. In recent years, graph neural networks (GNNs) have emerged as powerful models that leverage this graph structure and provide enhanced capacity for capturing complex and dynamic patterns within brain connectivity networks [4]. By allowing for end-to-end learning and automatic feature extraction from neuroimaging data, GNNs have opened new avenues for the study of connectivity in healthy and diseased states, enabling multimodal integration [5,6,7], dynamic connectivity analysis [6,8,9,10], and biomarker discovery [11,12].

While GNNs have shown significant promise in brain connectivity analysis, challenges remain. These include the integration of multimodal data, addressing the scarcity of labeled datasets, and developing interpretable models that allow insights into biological mechanisms [7,13,14,15]. Additionally, there is debate over the optimal strategies for modeling dynamic connectivity and the extent to which graph-based representations can encapsulate the full complexity of brain function. Nonetheless, current research has demonstrated the potential of GNNs to advance diagnostics, prognostics, and personalized interventions in neuroscience [5,7,8,13].

This paper reviews the current state of research in applying GNNs to brain connectivity, focusing on the methods, challenges, and future directions in this area. By examining recent advancements and identifying ongoing limitations, we aim to provide a comprehensive understanding of how GNNs can reshape the field of brain connectivity research, offering new perspectives on diagnostics and therapeutic strategies. Ultimately, this work highlights the transformative potential of GNNs and identifies key avenues for future research that may further advance their application in neuroscience.

## 2. Methodology

This review paper provides a comprehensive synthesis of the current literature on Graph Neural Networks (GNNs) in brain connectivity studies. The review was conducted by systematically gathering relevant peer-reviewed articles that address the intersection of GNNs and brain connectivity. The primary inclusion criteria for the articles were their focus on at least one of the following key themes: multimodal data integration, interpretability, and dynamic graphs. These three aspects were chosen because they represent critical areas of advancement and potential in GNN applications to brain connectivity.

**Figure 1:**
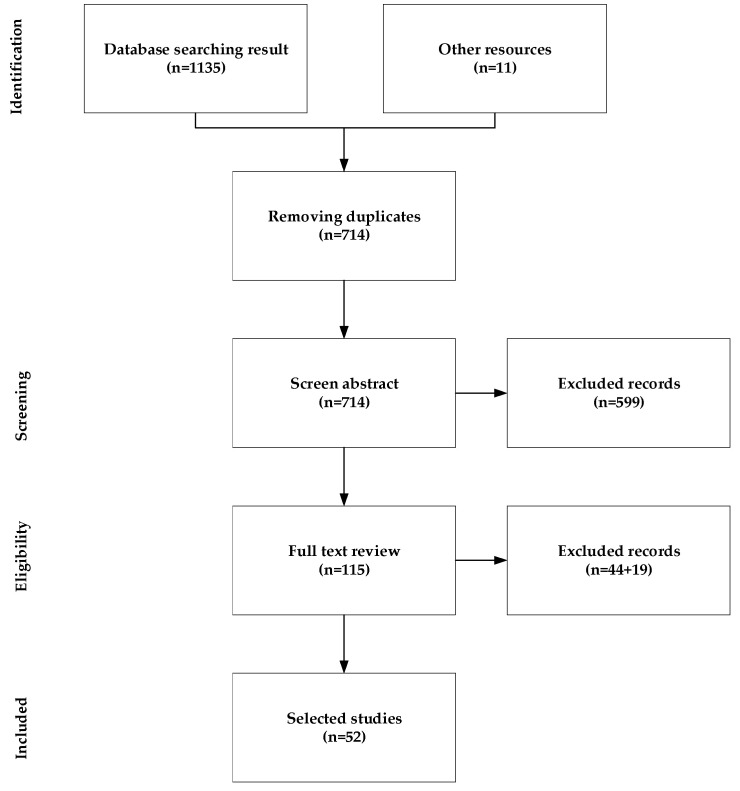
A flowchart illustrating the methodology and selection processes employed in this review, adhering to the PRISMA guidelines [16].

In the selection process, particular emphasis was placed on the novelty and relevance of the methodologies used in the studies, with a focus on those that introduced or applied GNN techniques specifically tailored to brain connectivity analysis. Studies that provided detailed insights into the practical application of GNNs for the analysis of fMRI or other brain imaging modalities, or those that explored the interpretability of GNN models, were prioritized. Articles that explored the temporal or dynamic aspects of brain connectivity networks using GNNs were also included, as these topics represent emerging and highly relevant areas of research. Figure 1 shows the whole process of article selection for this paper review.

### Data Extraction and Selection

The screening process involved the following steps:

1. Initial Search: To identify relevant literature, a thorough search was conducted across several well-established scientific databases, including PubMed, IEEE Xplore, Scopus, and Google Scholar. The search terms included combinations of “Graph Neural Networks”, “brain connectivity”, “multimodal”, “interpretability”, and “dynamic graphs”. A total of 1146 articles were identified during the initial search.
2. Title and Abstract Screening: Two researchers independently reviewed titles and abstracts to identify potentially relevant articles based on the inclusion criteria. Discrepancies were resolved through discussion. Only peer-reviewed papers published in reputable journals or conference proceedings were considered. A total of 432 articles were identified as duplicates and were excluded. Additionally, 599 articles were removed because they did not meet the inclusion criteria.
3. Full-Text Review: The full text of selected articles was examined to ensure that they met the inclusion criteria, particularly focusing on their use of GNNs for analyzing brain connectivity in the context of multimodal data, interpretability, or dynamic graphs. Forty-four articles were removed in this step.
4. Final Selection: Articles that met the inclusion criteria were included in the review. Data from these studies were extracted systematically, including information on study methods, datasets, key findings, and the specific application of GNNs. Finally, by removing another 19 articles, 52 articles remained for final consideration.

This process resulted in a final set of studies that were analyzed and summarized to identify trends, challenges, and future directions in the use of GNNs for brain connectivity analysis.

## 3. Preliminaries

### 3.1. Graph Definitions

A graph G=(V,E) is a mathematical structure that consists of a set of nodes V (or vertices) and edges E, where each edge represents a connection between pairs of nodes. In the context of brain imaging, each node can represent a specific region of interest (ROI) in the brain, and edges represent the relationships or connectivity (such as structural or functional) between these regions. Graphs can be either directed or undirected, weighted or unweighted, depending on the nature of the connections. For brain connectivity analysis, undirected and weighted graphs are commonly used, where the weights on edges represent the strength of connectivity between regions.

**Figure 2:**
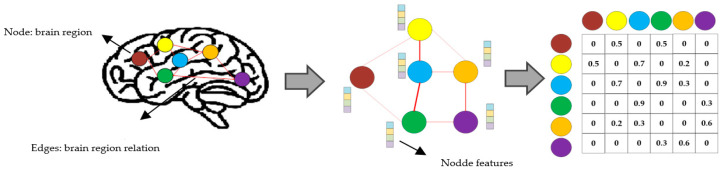
Flowchart illustrating the construction of a brain graph. Nodes represent distinct brain regions, while edges denote the relationships between these regions. Each node is associated with a feature vector, and edges may also have corresponding feature vectors. The adjacency matrix is subsequently derived from the graph to represent the structural connectivity between the brain regions.

Adjacency Matrix: The structure of a graph can be represented by an adjacency matrix A, where $A_{ij}$ indicates the presence (and possibly weight) of an edge between nodes i and j. For brain connectivity, the adjacency matrix often reflects the correlation or connectivity strength between different brain regions. Figure 2 shows basic graph construction for brain network and the adjacency matrix of the graph. Table 1 summarizes the key brain imaging modalities, their associated connectivity types, and the common metrics used for graph construction.

Laplacian Matrix: The Laplacian matrix L is another representation of a graph, defined as L=D−A, where D is the degree matrix (a diagonal matrix where each entry represents the number of connections for a node). The Laplacian matrix is particularly useful for spectral analysis in GNNs, helping capture smoothness over the graph structure.

### 3.2. Graph Neural Networks (GNNs)

Graph Neural Networks (GNNs) are deep learning architectures specifically designed for graph-structured data. Unlike traditional neural networks that work on grid-structured data (e.g., images or sequences), GNNs can learn patterns from data with arbitrary graph structures. A GNN typically consists of layers where each node aggregates features from its neighbors and updates its representation based on this aggregated information. This enables GNNs to capture the complex relationships and dependencies inherent in graph data.

Message Passing: A fundamental operation in GNNs is message passing, where each node aggregates feature information from neighboring nodes. For a given node v, the updated feature ${h_{v}}^{\left(k+1\right)}$ at layer k+1 can be computed as: $h_{v}^{\left(k+1\right)}=f\left(h_{v}^{\left(k\right)},\mathrm{AGGREGATE}\left(\left\{h_{u}^{\left(k\right)}:u\in N\left(v\right)\right\}\right)\right)$, where N(v) represents the neighbors of v, and f is a transformation function.

Popular GNN Variants: Various GNN architectures have been developed, each with different aggregation schemes. Common variants include Graph Convolutional Networks (GCNs), Graph Attention Networks (GATs), and GraphSAGE. Each variant has unique properties, making them suitable for different graph-based applications, including brain connectivity analysis. Table 2 provides a detailed summary of the various types of Graph Neural Networks (GNNs) applied in brain connectivity studies, highlighting their specific features and areas of application within this domain.

#### 3.2.1. Relationship Between Graph Neural Networks (GNNs) and Convolutional Neural Networks (CNNs)

**Figure 3:**
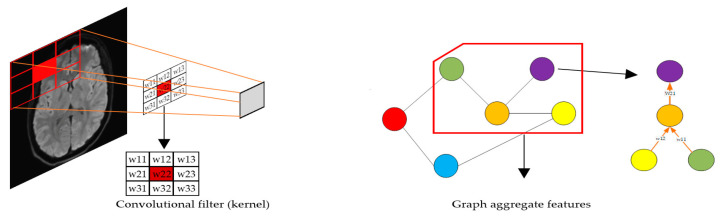
Comparison of localized information processing in Convolutional Neural Networks (CNNs) and Graph Neural Networks (GNNs). The left panel illustrates how CNNs aggregate information from a fixed, grid-like 3 × 3 patch of pixels using a convolutional filter. The right panel demonstrates how GNNs aggregate features from a node’s neighbors based on the graph’s adjacency structure, enabling learning on irregular, non-Euclidean data.

Graph Neural Networks (GNNs) are fundamentally inspired by Convolutional Neural Networks (CNNs) and extend their principles to graph-structured data. While CNNs excel at processing data that lie on regular, grid-like structures such as images, GNNs generalize these methods to non-Euclidean domains like graphs, where data points (nodes) are connected in an irregular structure through edges. This extension is achieved by reinterpreting the core operations of CNNs, such as convolution, parameter sharing, and feature aggregation, in a way that accommodates the unique properties of graphs. Some of the relations between GNN and CNN:

1. Neighborhood Aggregation: Adapting Convolution to Graphs
  At the heart of both CNNs and GNNs is the idea of localized information processing. In CNNs, a convolution operation involves sliding a filter (kernel) over small, fixed-sized regions of an image, aggregating pixel values within each region to detect patterns like edges or textures. GNNs adopt this concept by introducing a neighborhood aggregation mechanism, where each node updates its representation by combining information from its directly connected neighbors. This process is often referred to as “graph convolution”, which mimics the local receptive field of CNNs but operates on irregular graph structures. For instance, instead of aggregating values from a 3 × 3 patch of pixels, a node in a GNN aggregates features from its neighboring nodes based on the graph’s adjacency structure. This concept is visualized in Figure 3, which highlights the distinction between the two approaches.
2. Parameter Sharing: From Grids to Graphs
  The parameter-sharing principle in CNNs, where the same convolutional filter is applied across all spatial locations in an image, is also preserved in GNNs. In GNNs, the same aggregation rule and learnable parameters are shared across all nodes in the graph, ensuring scalability and consistency across graphs of varying sizes and structures. This parameter-sharing mechanism enables GNNs to efficiently learn graph-invariant features, just as CNNs learn translation-invariant features in images.
3. Pooling Techniques: Hierarchical Feature Representation
  Pooling, another crucial operation in CNNs, is similarly adapted in GNNs. In CNNs, pooling layers are used to down sample feature maps by summarizing information from local regions, thereby reducing computational complexity and emphasizing high-level features. In GNNs, graph pooling techniques achieve a similar effect by reducing the number of nodes in the graph while preserving its overall structure and important features. These adaptations make GNNs powerful tools for analyzing complex, irregular data that cannot be easily handled by traditional CNNs.
4. Mathematical Connection Between CNNs and GNNs

The relationship between CNNs and GNNs can also be illustrated mathematically. In CNNs, the convolution operation involves a weighted sum of pixel values within a fixed local region, producing hierarchical feature representations as the network deepens. In GNNs, this operation is generalized to the graph domain, where the feature of each node is updated by aggregating features from its neighbors, weighted by edge connections or learnable parameters. Both frameworks follow the same overarching principle of local feature aggregation, but GNNs adapt it to the complexities of graph topology. Mathematically, the operation is similar to CNNs but generalized:

$$\begin{array}{r} \mathrm{CNNs}:\;H_{i,j}^{\left(l+1\right)}=\sum_{k,l}W_{k,l}\cdot H_{i+k,j+l}^{\left(l\right)}\\ \mathrm{GNNs}:\;h_{v}^{\left(l+1\right)}=\sigma \left(\sum_{u\in N\left(v\right)}\frac{1}{\sqrt{d_{u}d_{v}}}W^{\left(l\right)}h_{u}^{\left(l\right)}\right) \end{array}$$

Here, $N\left(v\right)$ represents neighbors of v, and W plays the role of learnable filters, similar to CNNs.

#### 3.2.2. Key Components of GNNs for Brain Connectivity

**Figure 4:**
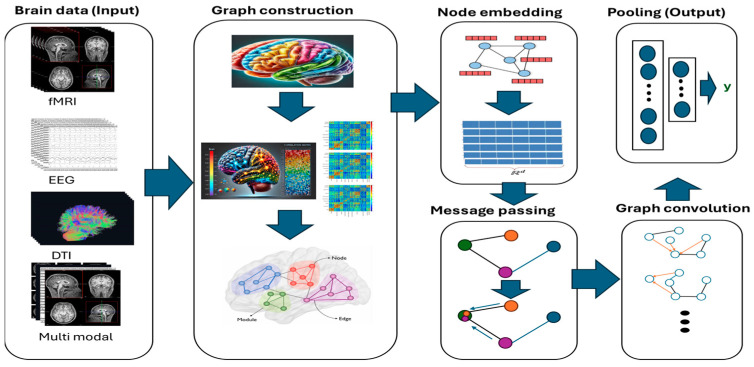
This figure illustrates a general framework for processing brain data using graph-based neural networks. It begins with various brain data modalities, including fMRI, EEG, DTI, and multi-modal data, which are transformed into graph representations. Each node in the graph represents a brain region or voxel, while edges represent connections between them. Node embeddings are then learned to represent each node’s features and relationships with other nodes. Finally, pooling operations, such as message passing or graph convolution, are employed to aggregate information from the graph and generate a final output.

To understand the role of GNNs in modeling brain connectivity, it is important to grasp the key components and operations that allow these models to process graph data effectively. As shown in Figure 4, we visualized the steps for analyzing brain data using graph neural networks.

Graph Construction: The first step in applying GNNs to brain connectivity is to construct the graph. Brain regions or voxels are typically treated as nodes, and their interactions—either structural or functional—are modeled as edges. For instance, in structural connectivity, edges can represent white matter fiber tracts derived from diffusion tensor imaging (DTI) data. In functional connectivity, edges represent the statistical dependencies between brain regions, such as those measured using resting-state fMRI.

Node Embeddings: GNNs learn to map the input features of nodes (e.g., the brain regions) into low-dimensional representations known as embeddings. These embeddings capture the underlying characteristics of each node, such as its connectivity with other regions, and serve as a basis for further learning tasks such as classification or prediction.

Message Passing Mechanism: GNNs rely on a message-passing paradigm, where nodes exchange information with their neighbors in the graph. This allows the model to incorporate not only local neighborhood information but also the global structure of the brain network. In the context of brain connectivity, this mechanism is key to understanding how the interactions between different brain regions contribute to overall brain function.

Graph Convolution Operations: A fundamental operation in GNNs is the graph convolution, where each node aggregates information from its neighbors to update its own feature representation. This operation is repeated across multiple layers, enabling the model to learn hierarchical representations of the brain network.

Pooling and Attention Mechanisms: Graph pooling techniques, such as global pooling or node-wise pooling, allow GNNs to aggregate information from multiple nodes and focus on the most important regions or edges in the brain network. Attention mechanisms, such as Graph Attention Networks (GATs), further enhance this by weighting the importance of different edges, making it possible to emphasize significant brain regions or connections in downstream tasks.

## 4. Brain Connectivity and Its Significance in Neuroscience

Brain connectivity refers to the intricate networks formed by neural circuits that interconnect various regions of the brain. Understanding brain connectivity is essential for elucidating the mechanisms underlying cognition, behavior, and neurological disorders. The brain is a highly complex organ where information is processed through dynamic interactions between distant regions, and these interactions are fundamental to cognitive functions such as perception, memory, and decision-making. Structural and functional connectivity are two critical aspects of brain connectivity, each providing complementary insights into the organization and functioning of the brain.

### 4.1. Structural Connectivity

Structural connectivity refers to the physical pathways that link different regions of the brain. These pathways are primarily composed of white matter tracts, which are bundles of axonal fibers that transmit signals between brain regions. Magnetic resonance imaging (MRI), particularly diffusion tensor imaging (DTI), is commonly used to map structural connectivity by visualizing the orientation and integrity of these white matter tracts. DTI allows researchers to study the directionality of water diffusion in the brain’s white matter, thereby providing valuable information on the brain’s structural wiring. The integrity of these white matter pathways is crucial for normal cognitive functioning, and disruptions in this connectivity are often associated with various neurological conditions, including neurodegenerative diseases such as Alzheimer’s and multiple sclerosis (MS) [15,17].

### 4.2. Functional Connectivity

On the other hand, functional connectivity refers to the temporal correlations between the activity of distinct brain regions. It is often studied using functional magnetic resonance imaging (fMRI), which measures changes in blood oxygenation level-dependent (BOLD) signals as a proxy for neuronal activity. Functional connectivity reflects the brain’s dynamic network organization during rest (resting-state fMRI, rs-fMRI) or while engaged in specific tasks. Unlike structural connectivity, which is relatively stable, functional connectivity can vary depending on the cognitive state and external stimuli. It has been shown that regions involved in similar cognitive processes tend to exhibit higher functional connectivity. Moreover, deviations in functional connectivity are associated with various psychiatric and neurological disorders, including depression, schizophrenia, and autism spectrum disorders [18].

### 4.3. Connectivity Applications

Both structural and functional connectivity provide complementary perspectives on the brain’s organization. While structural connectivity offers insights into the brain’s anatomical architecture, functional connectivity reveals the coordinated activity between brain regions that underlies cognition and behavior. The integration of these two aspects is crucial for understanding how the brain processes information across different scales and how disruptions in connectivity can lead to disease. For example, structural damage to white matter tracts in diseases such as Alzheimer’s can lead to altered functional connectivity, which may manifest as cognitive decline or memory impairment [19]. Therefore, examining both structural and functional connectivity is essential for gaining a comprehensive understanding of brain function and dysfunction.

In addition to its application in clinical research, brain connectivity is a powerful tool for understanding fundamental aspects of brain function, such as learning, adaptation, and development. For example, studies have demonstrated that the brain’s connectivity evolves across the lifespan, with significant changes in both structural and functional networks occurring from childhood through adulthood. These changes are thought to reflect maturational processes and the optimization of brain network configurations for efficient cognitive processing [18]. Similarly, understanding how brain networks are reorganized in response to learning and experience can provide insights into mechanisms of neuroplasticity and rehabilitation. Investigating how brain connectivity changes in response to different environmental, cognitive, and emotional experiences is a promising area of research, particularly for understanding resilience in the face of neurological or psychological stress.

The study of brain connectivity is also central to understanding individual differences in cognition and behavior. Variations in the organization and connectivity of the brain can explain why some individuals excel in specific cognitive tasks, while others may struggle. For instance, individual differences in working memory performance have been linked to differences in the connectivity between regions of the prefrontal cortex [20]. Likewise, differences in the connectivity between the default mode network and other brain regions have been associated with traits such as creativity, intelligence, and susceptibility to mental health conditions [21]. This personalized approach to brain connectivity research offers the potential for more tailored and effective interventions for individuals with neurological or psychiatric conditions.

### 4.4. Graph-Based Analysis in Brain Connectivity

Graph-based analysis has emerged as a powerful approach for modeling and studying brain connectivity due to its ability to represent complex, interconnected systems in a mathematically structured manner. In this context, the brain is conceptualized as a network, where individual brain regions or voxels serve as nodes and the connections between them—either structural or functional—are represented as edges. The application of graph theory to brain connectivity provides insights into the brain’s organizational principles, offering a more holistic understanding of how brain regions interact to support cognition and behavior.

Graph theory provides a formal framework for studying the topology of networks, including various key concepts such as nodes, edges, degree, centrality, and clustering. These concepts help quantify the network’s organization and its functional implications. The application of graph-based analysis to brain networks typically involves two main types of connectivity: structural connectivity and functional connectivity. Structural connectivity, as defined previously, pertains to the anatomical connections between brain regions, while functional connectivity refers to the temporal correlation of activity between those regions. Graph-based models can be applied to both types of connectivity, providing complementary perspectives on brain organization.

In the case of structural connectivity, brain regions are represented as nodes in the graph, and the connections (edges) between them are determined by white matter tracts. Diffusion tensor imaging (DTI) is commonly used to visualize and quantify the strength of these connections. On the other hand, functional connectivity networks are constructed based on the temporal correlations between activity in different brain regions, typically measured using resting-state fMRI (rs-fMRI). In functional brain networks, edges represent the correlation between the BOLD signal fluctuations of different brain regions, and nodes correspond to regions of interest (ROIs).

Graph-based analysis also allows for the quantification of global efficiency and local efficiency, which describe how efficiently information can be transmitted across the entire brain or within specific regions. Global efficiency measures the efficiency of communication across the entire network, while local efficiency focuses on the communication efficiency within a particular neighborhood of nodes. Both measures are critical for understanding how the brain integrates information at multiple scales. For example, disruptions in global efficiency have been observed in patients with neurodegenerative diseases, such as Alzheimer’s disease, where cognitive decline correlates with changes in the global structure of brain networks [22].

The application of graph-based models to brain networks is not limited to healthy individuals. In fact, it has proven particularly useful in investigating the pathophysiological mechanisms of various neurological and psychiatric disorders. For example, in Alzheimer’s disease, structural brain networks exhibit reduced small-worldness, global efficiency, and connectivity in critical regions such as the hippocampus, reflecting the disruption of information processing that underlies cognitive decline [23]. Similarly, in schizophrenia, alterations in the functional connectivity of brain networks, particularly in regions associated with working memory and executive function, have been identified through graph-based analysis. These findings have led to a better understanding of how specific network disruptions contribute to the cognitive deficits observed in these disorders [24].

Graph-based methods have also been used to explore dynamic connectivity, which refers to the time-varying nature of brain networks. While traditional static network models provide a snapshot of connectivity at a given time, dynamic network analysis allows for the study of how the brain’s connectivity changes over time. This approach is particularly useful for understanding how brain networks adapt to different cognitive tasks or rest states. Dynamic connectivity models have revealed that brain networks are not static but instead exhibit flexibility in their organization based on cognitive demands or environmental changes [25,26].

One of the most promising areas of research is the integration of multi-modal data using graph-based approaches. By combining structural, functional, and electrophysiological data, it is possible to construct more comprehensive models of brain connectivity. For example, the integration of structural MRI with functional MRI or electroencephalography (EEG) can provide a more nuanced understanding of how structural changes in the brain affect functional connectivity and vice versa. Such integrated models offer greater predictive power in understanding complex neurological conditions, such as epilepsy, autism, and major depressive disorder [27]. Multi-modal graph-based analysis allows for the study of these conditions from a network perspective, which could lead to more effective biomarkers and therapeutic targets. Table 3 shows an overview of common modality of brain data and corresponding types of connectivity in most of the papers.

## 5. Graph Neural Networks (GNNs) in Brain Connectivity

Graph Neural Networks (GNNs) have become a transformative tool in brain connectivity studies, particularly in the analysis of brain networks derived from various neuroimaging modalities. Traditional machine learning approaches are often limited by their inability to effectively handle the complex, non-Euclidean nature of brain connectivity data, where relationships between brain regions are best represented as graphs. GNNs, with their inherent ability to learn from graph-structured data, provide an elegant solution to this challenge, enabling the extraction of spatially and temporally meaningful representations from brain connectivity networks.

The architecture of GNNs is based on the premise of message passing, where nodes (representing brain regions) exchange information with their neighbors through edges (representing the connectivity between regions) in the graph. This process allows GNNs to capture both the local and global structural properties of the graph, such as community structure, connectivity patterns, and hierarchical organization. In the context of brain connectivity, GNNs can learn complex patterns in the data by aggregating features from neighboring brain regions, facilitating the detection of network abnormalities or the identification of disease biomarkers in neurological and psychiatric disorders [28].

### 5.1. GNN Advantages in Brain Connectivity

One of the key advantages of GNNs in the context of brain connectivity is their ability to incorporate multi-modal data. Brain connectivity is inherently multimodal, with different neuroimaging techniques providing complementary information about brain structure and function. GNNs can integrate data from multiple sources, such as structural MRI, functional MRI, and even electrophysiological data (e.g., EEG or MEG), to create a more comprehensive model of brain connectivity. This multi-modal integration enables the discovery of complex relationships between structural and functional networks, which might be overlooked when using a single modality in isolation. For instance, combining structural and functional connectivity through GNNs can help reveal how anatomical changes in the brain may lead to alterations in functional connectivity, providing deeper insights into the pathophysiology of neurological disorders [29,30].

Additionally, GNNs have shown great potential in the study of dynamic brain networks, which can capture the time-varying nature of brain connectivity. Dynamic connectivity, which refers to the temporal variability of brain activity patterns, is an emerging area of research. Traditional static graph models cannot fully capture these dynamic changes, whereas GNNs can model time-varying connectivity by incorporating temporal edges into the graph structure. This allows for the study of how the brain’s connectivity changes in real time and how these fluctuations may be associated with cognitive tasks or neurological conditions. Dynamic GNNs, which can process sequences of graphs, are particularly well-suited for analyzing fMRI data, where the connectivity between brain regions is not fixed but evolves over time in response to different stimuli or states [31,32].

In addition to structural and functional connectivity, GNNs can be used to address the challenge of inter-subject variability in brain network analysis. Different individuals exhibit considerable variation in their brain networks, and this variability must be accounted for in studies of brain connectivity. GNNs, with their ability to learn shared patterns across multiple subjects, can potentially identify common network features while accommodating individual differences. This makes GNNs particularly useful for studying large, heterogeneous populations, such as those with neurological disorders or during developmental studies, where brain connectivity may differ significantly across individuals. By leveraging the power of deep learning and graph-based approaches, GNNs can uncover group-level patterns that may be masked by individual variability.

The use of GNNs for biomarker discovery in brain connectivity is another promising area of research. Neurological and psychiatric disorders often involve disruptions to specific brain networks, and identifying these disruptions early on can lead to more effective interventions. GNNs have shown potential in identifying biomarkers for various conditions, including Alzheimer’s disease, schizophrenia, and autism spectrum disorders. By learning from large datasets of brain connectivity, GNNs can pinpoint subtle changes in brain networks that may serve as early indicators of disease. These biomarkers could potentially be used in clinical settings to assist with diagnosis, prognosis, and personalized treatment plans. For instance, in Alzheimer’s disease, GNNs have been used to identify altered patterns of brain connectivity that are associated with the disease’s progression, potentially allowing for early detection before overt clinical symptoms appear [27].

### 5.2. GNN Challenges in Brain Connectivity

One of the significant challenges in the application of GNNs to brain connectivity is the interpretability of the learned models. While GNNs are highly effective at capturing complex patterns, they are often seen as “black box” models, meaning that it can be difficult to understand exactly how they are making decisions. Interpretability is crucial in the neuroscientific domain, where the goal is not only to develop accurate models but also to gain insights into the underlying biological mechanisms. Recent advancements in explainable AI (XAI) techniques have started to address this issue by providing methods to visualize and interpret the learned representations of GNNs. For example, techniques like attention mechanisms or saliency maps can be used to highlight the most important nodes and edges in the brain network that contribute to a particular prediction or classification, offering more transparency in the model’s decision-making process [30,31,32,33].

Beyond interpretability, there are several other disadvantages of using GNNs for brain connectivity analysis:

1. Data Preprocessing and Graph Construction: Constructing appropriate graphs from brain imaging data is a challenging task. Brain networks are complex, with varying levels of connectivity between regions, and determining which features to include as nodes and edges can be difficult. Additionally, the construction of dynamic graphs, especially when analyzing time-series fMRI data, is computationally expensive and may introduce noise if temporal resolutions are not aligned correctly.
2. Scalability: GNNs, particularly those involving large, high-dimensional brain networks, can struggle with scalability. The number of nodes (brain regions) and edges (connectivity patterns) can be vast, leading to significant memory and computational resource requirements. As the size of the brain network grows, the complexity of the GNN increases, potentially leading to slower processing times and a higher risk of overfitting.
3. Overfitting and Generalization: Due to the relatively small sample sizes common in brain connectivity studies, there is a risk of overfitting when using GNNs. Brain data are often sparse, with many features or edges missing, and training on limited datasets may result in models that do not generalize well to unseen data. This issue is especially relevant when using complex models like GNNs, which have a large number of parameters relative to the available data.
4. Handling Heterogeneity: The human brain exhibits significant individual variability, meaning that brain connectivity patterns can differ widely across individuals. This heterogeneity poses a challenge for GNN models, as they need to generalize across different brain structures while accounting for these variations. Developing a GNN that can adapt to individual differences in brain connectivity remains an ongoing research challenge.
5. Long Training Times: GNNs can require extensive training times, particularly when dealing with large datasets or complex architectures. This is especially problematic in the context of brain connectivity studies, where data may need to be processed from multiple brain regions across different individuals, and training must account for the temporal and spatial dynamics of the brain. The time-consuming nature of training these models can limit their practical application, especially in clinical or real-time settings.

## 6. Recent Advances in GNNs for Brain Connectivity Modeling

Graph Neural Networks (GNNs) have significantly enhanced the ability to model brain connectivity, facilitating new insights into the complex structure and function of the brain. The advances have been driven by the increasing availability of large neuroimaging datasets, improvements in computational resources, and innovations in GNN architectures. As a result, GNNs have emerged as a powerful tool for analyzing brain connectivity, enabling the integration of diverse data modalities, capturing dynamic changes in brain networks, and improving the interpretability of models. This section discusses some of the most notable advances in GNNs for brain connectivity modeling, with a focus on novel techniques, applications, and the challenges that remain to be addressed. Table 4 and Table 5 present a comprehensive summary of the selected studies. Table 4 outlines the data types, connectivity types, and study objectives, while Table 5 highlights the key findings, performance metrics, and areas of novelty in these studies.

### 6.1. Capturing Time Variant of Brain Data

One of the most significant advances in GNNs for brain connectivity is the development of dynamic GNN models capable of capturing the time-varying nature of brain networks. Traditional methods in brain connectivity analysis, such as static graph representations of functional or structural networks, fail to account for the dynamic changes in connectivity that occur over time. However, in the context of functional connectivity, brain activity is inherently dynamic, fluctuating in response to cognitive tasks, resting states, and other factors. To address this, recent studies have employed dynamic GNNs that integrate temporal edges into the graph structure. These models allow the network to evolve over time, capturing fluctuations in brain connectivity that may be linked to cognitive processes, disease states, or other neurological phenomena [9,15]. These dynamic models can be particularly useful for studying the brain’s response to external stimuli or tracking the progression of neurodegenerative diseases where connectivity changes over time. By leveraging time-series data, dynamic GNNs have the potential to reveal how brain connectivity evolves and how these changes are related to specific behaviors or clinical outcomes.

### 6.2. Multi Modal Brain Data Analysis

A related advancement is the integration of multi-modal brain imaging data into GNN models. The brain is a highly complex organ, and a comprehensive understanding of its connectivity requires data from various neuroimaging modalities, such as structural MRI, functional MRI (fMRI), diffusion tensor imaging (DTI), and electrophysiological data like EEG or MEG, and even non-imaging data such as demographic and clinical assessments data. Recent developments in GNNs have focused on the simultaneous processing of multiple modalities, enabling the construction of multi-modal brain networks that capture the interplay between structural and functional connectivity. For example, researchers have developed methods that fuse structural brain graphs derived from DTI with functional brain graphs from resting-state fMRI to better understand how anatomical connections influence functional interactions between brain regions [40,45,46]. By incorporating complementary information from different modalities, multi-modal GNNs provide a more holistic view of brain connectivity, facilitating a deeper understanding of the brain’s organizational principles and how they are altered in disease.

### 6.3. Interpretability

Another important advancement is the use of graph attention mechanisms within GNNs, which aim to improve the interpretability and performance of the models. Traditional GNNs treat all neighboring nodes in a graph equally when aggregating information, but this approach may not be optimal in the case of brain networks, where some brain regions have stronger or more significant interactions than others. Attention mechanisms allow the model to focus on the most important neighbors, giving higher weights to edges or nodes that contribute more meaningfully to the overall connectivity pattern. This approach has been particularly valuable in brain connectivity modeling, where it enables GNNs to emphasize key brain regions or connections that may be central to a particular cognitive function or pathology. For example, attention-based GNN models have been shown to improve the accuracy of brain disease classification tasks by highlighting regions that are most predictive of conditions such as Alzheimer’s disease or schizophrenia [15,41]. These attention mechanisms not only enhance model performance but also provide valuable insights into the brain regions that are most relevant for understanding specific diseases.

In parallel with attention mechanisms, graph convolutional networks (GCNs) have been widely adopted for brain connectivity modeling. GCNs allow for the aggregation of node features based on their neighboring nodes, providing a robust way to learn representations of brain regions that account for their structural and functional relationships. Recent work has focused on improving the scalability and efficiency of GCNs, enabling them to process large-scale brain networks with thousands of nodes, as is typical in neuroimaging studies. These advancements have facilitated the use of GCNs in the analysis of whole-brain networks, where individual regions or voxels are treated as nodes, and the edges represent connectivity measures such as functional correlation or structural connectivity. By using GCNs, researchers have been able to analyze large, high-dimensional datasets, improving our understanding of complex brain networks and their alterations in various neurological conditions [29,34,47,48,49,50]. These advancements are particularly useful in the study of large patient cohorts, where individual variability must be accounted for to identify generalizable patterns in brain connectivity.

### 6.4. Semi-Supervised Learning

The application of semi-supervised learning techniques in GNNs has also gained traction in the field of brain connectivity modeling. Semi-supervised learning methods leverage both labeled and unlabeled data to improve model performance, which is especially useful in the context of brain imaging, where labeled data (such as disease labels or clinical outcomes) are often limited. By using a graph structure that encodes both labeled and unlabeled nodes, semi-supervised GNNs can learn to classify or predict outcomes based on the relationships between brain regions, even when only a small portion of the data are labeled. This approach has been applied to various brain disorders, where GNNs can learn to differentiate between healthy and pathological brain networks with limited clinical annotations, thus improving the generalizability of models to new subjects or populations [51,52].

## 7. Challenges and Limitations of GNNs in Brain Connectivity Studies

While Graph Neural Networks (GNNs) have demonstrated considerable promise in brain connectivity studies, several challenges and limitations persist in their application to neuroimaging data. These challenges encompass technical, methodological, and conceptual issues, which must be addressed for GNNs to reach their full potential in understanding brain networks and their alterations in neurological conditions. This section discusses some of the key obstacles in the deployment of GNNs for brain connectivity analysis, including data-related issues, model interpretability, scalability, and generalization across populations.

### 7.1. Variability of Neuroimaging Data

One of the most significant challenges in applying GNNs to brain connectivity studies is the heterogeneity and variability of neuroimaging data. Neuroimaging data, particularly from brain networks, exhibit substantial variability across individuals due to differences in anatomy, functional organization, and connectivity patterns. This variability poses difficulties in constructing meaningful and consistent brain graphs that can be used for GNN analysis. Moreover, the sparsity of brain connectivity graphs is another issue, as the number of connections between brain regions in individual subjects is often limited. In fMRI data, for instance, the presence or absence of functional connections between regions may vary across subjects, and this variability can complicate the modeling process. To address these challenges, preprocessing techniques such as normalization, registration, and spatial smoothing are commonly employed, but these methods may introduce bias or fail to capture certain critical features of the data. Additionally, the identification of appropriate connectivity measures (e.g., correlation, coherence, or synchronization) to define the edges of brain graphs remains an open question, as different metrics may lead to different conclusions regarding the organization of brain networks [15,53].

### 7.2. Scalability

Another key limitation is the scalability of GNNs when applied to large-scale brain networks. Brain networks often consist of thousands of nodes, with each node representing a brain region or voxel, and the number of edges between nodes can be vast. Traditional GNN architectures struggle to efficiently process such high-dimensional graphs, as the computational complexity grows rapidly with the size of the network. While various solutions have been proposed to improve the scalability of GNNs, such as graph sampling, hierarchical pooling, or graph coarsening, these approaches often introduce trade-offs in model accuracy or lose fine-grained details of the connectivity structure. In the context of brain connectivity, retaining sufficient detail is crucial, as small changes in connectivity patterns can have important clinical implications, particularly in the study of neurological diseases like Alzheimer’s disease, schizophrenia, or epilepsy. Moreover, large-scale neuroimaging datasets often exceed the memory capacity of standard computing resources, which necessitates the use of specialized hardware or distributed computing methods. However, even with these resources, efficient scaling of GNNs remains a challenging task, particularly in studies involving large patient cohorts or longitudinal datasets.

### 7.3. Brain Data Dimensions

A related challenge is the dimensionality of brain connectivity data, which often exceeds the capacity of traditional machine learning methods. Brain connectivity matrices derived from functional or structural neuroimaging data can be high-dimensional, with hundreds or even thousands of features (i.e., brain regions or voxels) representing each subject. This high dimensionality can lead to overfitting, especially when the number of available training samples is relatively small, as is often the case in clinical neuroimaging studies. Although GNNs are designed to handle sparse, high-dimensional data, they still face difficulties in learning robust representations when the number of nodes is large relative to the number of labeled samples. Regularization techniques, such as dropout, weight decay, or early stopping, can help mitigate overfitting, but selecting the right combination of regularization strategies for a specific dataset remains an open problem. Furthermore, the high dimensionality of brain data can result in the curse of dimensionality, making it difficult to generalize models across different datasets or populations. This challenge is particularly pertinent in studies that aim to build predictive models for clinical decision-making, where accurate generalization to unseen subjects or populations is essential.

### 7.4. Lack of Interpretability

Another major limitation of GNNs in brain connectivity research is the lack of interpretability of the learned models. While GNNs excel at learning complex, high-dimensional representations, they often function as black-box models, providing limited insight into the underlying mechanisms driving the observed connectivity patterns. In brain connectivity studies, it is crucial to understand which brain regions or network connections are contributing to the model’s predictions, especially when applied to clinical populations. For example, in the context of Alzheimer’s disease, a GNN may be able to classify patients based on their brain connectivity profiles, but without an interpretable model, it is unclear which specific regions or network alterations are most indicative of disease progression. Interpretability is especially important when GNNs are used in clinical settings, where clinicians need to understand the rationale behind the model’s predictions to make informed decisions. To address this, recent research has explored methods for increasing the transparency of GNNs, such as the use of attention mechanisms, saliency maps, or feature importance scores, but these techniques are still in early stages of development. Thus, achieving reliable and interpretable models remains an important goal in brain connectivity research.

### 7.5. Generalization of GNN Models

The generalization of GNN models across different datasets or populations represents another critical challenge. Neuroimaging data are highly subject-dependent, and variability in brain structure, function, and connectivity across populations can undermine the generalizability of GNN models. For example, models trained on a cohort of healthy individuals may not perform well when applied to clinical populations or individuals from different demographic backgrounds, such as age, sex, or genetic predisposition.

Additionally, the protocols used in different neuroimaging studies, such as variations in scanning parameters, acquisition techniques, and data preprocessing methods, can lead to systematic differences in the data that affect model performance. While transfer learning and domain adaptation techniques have been proposed to improve the generalization of GNNs across different datasets, these methods are still under active investigation and have not yet been widely adopted in brain connectivity research. Furthermore, the lack of large, diverse datasets with comprehensive clinical annotations limits the ability to develop and validate generalizable GNN models. Efforts to curate multi-site, multi-population datasets will be critical to overcoming this challenge and ensuring that GNN models are applicable to real-world clinical scenarios.

### 7.6. Interpretation of Causality Versus Correlation

Finally, a significant challenge in GNN-based brain connectivity research is the interpretation of causality versus correlation in brain networks. Many studies use GNNs to identify associations or patterns in brain connectivity, but distinguishing between causally relevant connections and mere correlations remains a complex task. In neuroscience, establishing causal relationships between brain regions is often difficult due to the observational nature of neuroimaging data. Although GNNs can uncover associations in brain networks, they do not inherently provide a framework for determining causal links between regions or networks. Approaches that integrate GNNs with other methods, such as causal modeling or reinforcement learning, may offer more insight into the causal mechanisms underlying brain connectivity. However, these approaches are still in the early stages of development and require further validation in the context of brain connectivity research.

While GNNs have shown substantial promise in advancing the analysis of brain connectivity, several challenges and limitations must be addressed for their widespread adoption in neuroscience. These include the high variability and sparsity of neuroimaging data, issues related to model scalability and generalization, the complexity of interpretability, and the difficulty in establishing causal relationships. As advancements in computational techniques, model design, and data collection continue to progress, many of these challenges can be overcome, further enhancing the utility of GNNs in brain connectivity studies and their application to clinical neuroscience.

## 8. Future Directions in GNNs for Brain Connectivity

The application of Graph Neural Networks (GNNs) to brain connectivity is an emerging area of research with immense potential to advance our understanding of brain organization and its alteration in neurological disorders. Despite the significant progress made in the field, several avenues for future exploration remain, with promising developments in computational techniques, model refinement, and the integration of multi-modal data. This section outlines potential future directions in GNNs for brain connectivity, emphasizing areas such as the incorporation of longitudinal data, improving model interpretability, advancing computational efficiency, integrating multi-modal neuroimaging, and leveraging clinical translation.

### 8.1. Use of Longitudinal Neuroimaging Data

One of the most exciting areas for future research is the use of longitudinal neuroimaging data to study brain connectivity changes over time. Longitudinal studies allow for the tracking of dynamic changes in brain networks and the identification of early biomarkers for neurodegenerative diseases such as Alzheimer’s disease, Parkinson’s disease, and Huntington’s disease. However, analyzing temporal data with GNNs remains a challenge due to the need to model both the spatial and temporal dimensions of connectivity. Traditional GNNs are static, meaning they treat the brain network as a fixed structure, which limits their ability to capture the dynamic changes in connectivity over time. To address this, future work should focus on the development of temporal or dynamic GNN models that can learn time-varying graph representations and capture the evolving nature of brain networks. Techniques such as recurrent neural networks (RNNs) or temporal message passing could be incorporated into GNN frameworks to handle the complexities of longitudinal data and improve their predictive capabilities. Such models could be instrumental in understanding the progression of neurological diseases, identifying critical time windows for intervention, and predicting disease trajectories.

### 8.2. Enhancement of Model Interpretability

Another key direction for future research is the enhancement of model interpretability. As GNNs become more widely used in brain connectivity studies, it is essential to ensure that their results are interpretable, particularly when applied to clinical contexts. Current GNN models often operate as black boxes, providing accurate predictions but offering limited insight into the underlying neural mechanisms driving these predictions. Interpretability is especially crucial in the context of clinical neuroscience, where understanding the role of specific brain regions or network alterations can guide treatment strategies and improve patient outcomes. Future research should explore methods to make GNN models more transparent, such as by incorporating attention mechanisms, which allow the model to focus on the most relevant regions or connections in the brain, or by developing saliency maps that highlight the important features contributing to model decisions. Additionally, the integration of explainable AI (XAI) techniques could provide a framework for interpreting complex neural network models, making them more accessible and understandable to clinicians. Advances in interpretability will foster greater trust in GNN models and facilitate their adoption in clinical settings.

### 8.3. Cope with Scalability Challenges

The computational scalability of GNNs remains a significant challenge, particularly when applied to large-scale brain networks derived from high-resolution neuroimaging data. As neuroimaging technology improves, datasets will continue to grow in size and complexity, posing challenges for traditional GNN architectures. Future directions should focus on the development of more efficient GNN models that can handle the large, sparse graphs typically found in brain connectivity studies. Techniques such as graph sparsification, which reduces the number of edges in the graph while retaining critical connectivity information, or graph coarsening, and multi-resolution approaches could be explored to improve the scalability of GNNs. Additionally, parallel computing and the use of specialized hardware such as graphics processing units (GPUs) and tensor processing units (TPUs) will be crucial for processing large-scale datasets in a timely manner. Furthermore, advances in distributed learning frameworks could allow GNN models to be trained across multiple machines, improving efficiency and reducing the computational burden.

### 8.4. Multi-Modal Data Integration

The integration of multi-modal neuroimaging data represents another exciting direction for future work. Brain connectivity is influenced by multiple aspects of brain structure and function, which can be captured through a variety of neuroimaging modalities such as structural MRI, functional MRI (fMRI), diffusion tensor imaging (DTI), magnetoencephalography (MEG), and electroencephalography (EEG). Each of these modalities provides complementary information about the brain and integrating them into a unified model could provide a more comprehensive understanding of brain networks and their alterations in disease. For instance, structural MRI provides detailed information about brain anatomy, while fMRI captures dynamic functional connectivity patterns. DTI, on the other hand, can provide insight into white matter connectivity. GNNs are particularly well-suited for multi-modal data integration because of their ability to model complex relationships between different types of data. Future research should focus on developing multi-modal GNN architectures that can fuse information from different neuroimaging techniques, leveraging the complementary strengths of each modality. For example, joint embedding spaces or multi-stream networks could be used to simultaneously model structural and functional connectivity in brain networks. The integration of multi-modal data could lead to more accurate and robust brain network models, enhancing our understanding of how different aspects of brain connectivity contribute to cognition, behavior, and disease.

### 8.5. Clinical Translation

Moreover, clinical translation of GNN models is an area that warrants significant attention. While GNNs have shown promise in experimental settings, their integration into real-world clinical practice remains a challenge. One of the key obstacles is the need for GNN models to generalize across diverse populations, including patients with varying clinical characteristics, such as age, sex, and comorbidities. Models trained on data from one cohort may not perform well when applied to other cohorts, limiting their clinical applicability. Future work should focus on developing generalizable GNN models that can be applied across diverse populations and neuroimaging protocols. This could involve the use of techniques such as transfer learning and domain adaptation, which allow models to be fine-tuned on new datasets with limited labeled data. Additionally, the development of standardized protocols for neuroimaging acquisition and data preprocessing would help ensure that GNN models are trained on consistent, high-quality data, further improving their clinical utility. Ultimately, the goal is to develop GNN-based diagnostic tools that can assist clinicians in identifying biomarkers, predicting disease progression, and personalizing treatment strategies for patients with neurological disorders.

## 9. Conclusions

Graph Neural Networks (GNNs) have shown significant promise in advancing brain connectivity analysis, enabling a more nuanced understanding of the human brain’s complex network dynamics. By leveraging GNNs, researchers are now able to model intricate, non-linear relationships in brain networks, integrate multimodal imaging data, and explore dynamic connectivity patterns that are essential for capturing real-time brain activity. These capabilities represent an important shift from traditional graph-theoretical approaches, facilitating improved diagnostics, prognostics, and personalized therapeutic strategies for neurological disorders. Despite these advances, substantial challenges persist, particularly in the areas of model interpretability, data scarcity, and robust multimodal integration, which restrict the widespread clinical adoption of GNNs. Addressing these challenges will be essential for realizing the full potential of GNNs in neuroscience. Future research should focus on developing more interpretable models, improving data access through collaborative efforts, and refining multimodal GNN architectures to achieve a comprehensive understanding of brain connectivity patterns. This continued innovation will pave the way for more accurate and individualized insights into brain health, offering the potential to transform both research and clinical approaches in the field of brain connectivity studies.

## Figures and Tables

**Table 1:** Brain Imaging Modalities, Connectivity Types, and Graph Construction Metrics.

Modality	Description	Connectivity Type (Structural, Functional, Dynamic)	Common Metrics/Representations
MRI	Structural brain imaging	Structural	Adjacency matrices, fiber tracts
fMRI	Functional brain imaging	Functional, Dynamic	Pearson correlation, coherence
EEG/MEG	Electrophysiological signals	Functional, Dynamic	Cross-spectral densities, coherence
PET	Positron Emission Tomography	Functional	Glucose metabolism, regional cerebral blood flow
DTI	Diffusion Tensor Imaging	Structural	Fractional anisotropy (FA), tractography, connectivity matrices
Multimodal	Combined imaging modalities	Structural, Functional, Dynamic	Hybrid metrics

**Table 2:** Overview of different types of Graph Neural Networks (GNNs) utilized in brain connectivity studies.

GNN Variant	Key Features	Common Applications in Brain Studies
GCN (Graph Convolutional Networks)	Laplacian-based filters, spectral domain	Static connectivity classification
GAT (Graph Attention Networks)	Attention mechanisms for edge weighting	Regional feature importance studies
DGCNN (Dynamic Graph CNNs)	Temporal graph analysis	Dynamic connectivity studies
Hierarchical GNNs (Hier GNN)	Multi-level graph structures, hierarchical learning	Multiscale brain connectivity analysis
Spatiotemporal GNNs	Integration of spatial and temporal data	Spatiotemporal brain network analysis

**Table 3:** Overview of Brain Imaging Modalities and Connectivity Types.

Modality	Description	Connectivity Type	Common Metrics/Representations
MRI	Structural brain imaging	Structural	Adjacency matrices, fiber tracts
fMRI	Functional brain imaging	Functional, Dynamic	Pearson correlation, coherence
DTI	Diffusion Tensor Imaging	Structural	Fractional anisotropy (FA), tractography, connectivity matrices
EEG/MEG	Electrophysiological signals	Functional, Dynamic	Cross-spectral densities, coherence
Multimodal	Combined imaging modalities	Structural, Functional, Dynamic	Hybrid metrics

**Table 4:** Overview of Data Types, Connectivity Types, and Objectives in Selected Studies.

Study	Year	GNN Variant	Data Type	Type of Connectivity	Objective
Li et al. [15]	2021	GCN	fMRI	Functional	Identify neurological biomarkers and improve fMRI analysis
Wein et al. [6]	2021	DGCNN	DTI and fMRI	Structural and Effective Connectivity	Integrate fMRI and DTI data to infer causal dependencies and study the structure-function relationship in the brain
Sebenius et al. [34]	2021	Hier GNN	Structural and Functional MRI	Structural and Functional	Develop a multimodal GNN (MM-GNN) framework to integrate structural and functional connectivity for schizophrenia diagnosis.
Kim et al. [10]	2021	Spatio-Temporal Attention GNN	fMRI	Functional	Learn dynamic graph representation of brain connectome using spatio-temporal attention. Address dynamic FC network limitations in GNNs.
Hu et al. [35]	2021	GAT	fMRI	Functional	To classify ASD versus HC brain networks and interpret feature importance.
Cui et al. [36]	2022	GCN	fMRI	Structural and Functional	Develop an interpretable GNN framework (IBGNN) to predict brain disorders and identify disorder-specific biomarkers.
Zhu et al. [37]	2022	GCN	DTI, fMRI	Structural and Functional	Develop a GNN framework using contrastive learning for jointly embedding multimodal brain networks with message passing.
Zhdanov et al. [38]	2022	GCN, GAT	EEG	Functional	Develop a GNN classifier to differentiate mental states and schizophrenia subtypes using EEG data; apply GNNExplainer for interpretability.
Li et al. [9]	2022	GAT	EEG	Functional	Detect and classify epileptic seizures, and dynamically analyze brain functional connectivity
Zhang et al. [5]	2023	GAT	fMRI and phenotypic	Functional	Classify ASD and AD using local/global brain features
Tong et al. [39]	2023	GCN	fMRI	Functional	Propose a SFC-GNN for brain disease diagnosis using fMRI data by combining graph convolution and node pooling layers.
Zhang et al. [40]	2023	GCN	sMRI, PET, Phenotypic data	Structural and functional connectivity	Early diagnosis of Alzheimer’s disease (AD) using multi-modal imaging and phenotypic data
Wang et al. [12]	2023	GAT	fMRI, Gene Expression	Functional	ASD diagnosis and biomarker discovery
Zheng et al. [7]	2024	Hier GNN	fMRI and synthetic data	Functional	To develop an interpretable GNN model for brain network-based psychiatric diagnosis by identifying causally related subgraphs.
Yang et al. [8]	2024	DGCNN	fMRI, EEG, DTI	Multi-modal connectivity	Propose a HyperComplex Graph Neural Network (HC-GNN) for the integration and analysis of multi-modal brain networks.
Wang et al. [11]	2024	GAT	fMRI	Functional, Interaction-based Connectivity	Classify brain disorders using multimodal GNNs and identify biomarkers.
Zheng et al. [41]	2024	GCN	fMRI	Functional	Psychiatric disorder diagnosis, subtyping, and biomarker discovery
Moon et al. [42]	2024	GAT	Diffusion MRI, Behavioral data	Structural	Brain age estimation and identification of neural connections associated with Alzheimer’s risk
Wang et al. [43]	2024	GCN	rs-fMRI	Functional	To optimize GNN architectures for schizophrenia spectrum disorder prediction.
Amendola et al. [44]	2024	GCN, GAT	MRI	Spatial	To evaluate GNN effectiveness and improve model explainability for brain tumor segmentation.

**Table 5:** Key Findings, Performance Metrics, and Novel Contributions of Selected Studies.

Study	Year	Key Findings	Performance Metrics	Novelty Area
Li et al. [15]	2021	Outperformed alternatives, detected salient ROIs, aligned with prior evidence for ASD biomarkers and task states	accuracy, F1-score, recall, and precision	Interpretability
Wein et al. [6]	2021	Demonstrated ability of GNNs to capture long-term dependencies, outperforming VAR in causal inference.	Accuracy, scalability, and generalizability across protocols	Multimodal and Dynamic
Sebenius et al. [34]	2021	Multimodal pooling dramatically improves classification performance compared to single-modal and non-GNN baselines, while providing interpretable and biologically plausible ROI salience.	Classification Accuracy (mean AUC)	Multimodal, Interpretability
Kim et al. [10]	2021	STAGIN integrates temporal sequences of brain graphs for dynamic representation with spatial and temporal explainability. Achieves high performance in both resting-state and task fMRI datasets.	Accuracy, compared with static FC	Dynamic
Hu et al. [35]	2021	GAT-LI, using the GAT2 model, outperformed other models on ABIDE I database, achieving the best classification performance. GNNExplainer was superior to Saliency Map and DeepLIFT in identifying critical features.	Accuracy, sensitivity, F1	Interpretability
Cui et al. [36]	2022	Group-level interpretation of shared biomarkers across subjects and improved disorder prediction performance using an explanation generator combined with the backbone model.	Classification accuracy, biomarker interpretability, robustness to individual image quality	Interpretability
Zhu et al. [37]	2022	Effectively fused multimodal brain networks; introduced degree-based node features and edge-aware message passing for enhanced node embeddings.	Classification accuracy, multimodal embedding consistency	Multi modal
Zhdanov et al. [38]	2022	Demonstrated state-of-the-art performance for EEG classification and validated GNNExplainer results with domain knowledge of brain connectivity during dichotic listening tasks.	Classification accuracy, model interpretability	Interpretability
Li et al. [9]	2022	Achieved 91% accuracy in classifying 7 types of seizures; outperformed CNN (65%), standard GNN (74%), and transformers (82%); proposed dynamic spatial-temporal graph generation for EEG signals	Macro F1, Weighted F1	Dynamic
Zhang et al. [5]	2023	Identified biomarkers, achieved SOTA performance	classification accuracy (Acc), area under the curve (AUC), sensitivity (Sen), and F1-score	Multimodal
Tong et al. [39]	2023	SFC-GNN effectively selects important brain regions for classification, reducing computational time and providing interpretable results for disease diagnosis.	two-node importance	Interpretability
Zhang et al. [40]	2023	Proposed a multi-modal GNN framework combining node vectors and adjacency matrices for improved AD diagnosis; demonstrated that phenotypic data enhances diagnostic accuracy	accuracy (ACC), sensitivity (SEN), specificity (SPE), and the area under curve (AUC)	Multi modal
Wang et al. [12]	2023	Achieved an accuracy of 79.78%; identified critical brain regions (social-brain circuit, default-mode network, sensory perception network) and ASD-related genes (MUTYH, AADAT, MAP2); links between functional connectivity and gene expression were established	Accuracy	Multi modal
Zheng et al. [7]	2024	CI-GNN outperforms baseline GNNs and state-of-the-art explainers in accuracy and interpretability, providing concise, clinically meaningful explanations for psychiatric diagnosis.	Accuracy, explainability, reliability, causal validity (via Conditional Mutual Information constraint)	Multimodal, Interpretability
Yang et al. [8]	2024	HC-GNN enhances the fusion of heterogeneous modalities (e.g., fMRI, EEG, DTI) by modeling them as hypercomplex tensor graphs, achieving superior classification performance and scalability across multiple datasets.	Classification performance, scalability, saliency map analysis	Multi modal, Dynamic
Wang et al. [11]	2024	Achieved 80.66% accuracy; discovered novel biomarkers linked to autism, validated by literature.	Accuracy	Multi modal
Zheng et al. [41]	2024	BPI-GNN effectively discriminated psychiatric patients (MDD, ASD) from healthy controls and identified biologically meaningful subtypes with clinical and gene expression relevance. Outperformed 11 popular methods	F1-score and Matthew’s Correlation Coefficient (MCC)	Interpretability
Moon et al. [42]	2024	FAGNN accurately estimated brain age, identified significant brain regions and connections (e.g., cingulum, corpus callosum, hippocampus), and highlighted APOE genotype and diet as modulators of brain aging. Key connections were validated using diffusion MRI metrics.	MAE, RMSE	Multi modal
Wang et al. [43]	2024	Proposed EA-GNAS improves diagnosis of schizophrenia with enhanced accuracy, F1-score, and AUC. Model predictions were explained using GNNExplainer, revealing important brain regions in SSD.	Accuracy, precision, recall, F1	Interpretability
Amendola et al. [44]	2024	GNN models achieved precise tumor segmentation on BraTS 2021 data. GNNExplainer enhanced model interpretability by identifying key edges and features contributing to segmentation decisions, advancing transparency in clinical applications.	Adaptive segmentation accuracy (ASA)	Interpretability
